# Role of phosphodiesterase inhibitor Ibudilast in morphine-induced hippocampal injury

**DOI:** 10.5249/jivr.v6i2.497

**Published:** 2014-07

**Authors:** Mohsen Zhaleh, Marzieh Panahi, Mehri Ghafurian Broujerdnia, Rostam Ghorbani, Kambiz Ahmadi Angali, Ghasem Saki

**Affiliations:** ^a^Department of Anatomy, Ahvaz Jundishapur University of Medical Sciences, Ahvaz, Iran.; ^*b*^Immunology Department, Fertility and Infertility Research Center, Ahvaz Jundishapur University of Medical Sciences, Ahvaz, Iran.; ^*c*^Department of Anatomy and Cell Biology, Kermanshah University of Medical Sciences, Kermanshah, Iran.; ^*d*^Department of Epidemiology and Statistics, Faculty of Public Health , Ahvaz Jundishapur University of Medical Sciences, Ahvaz, Iran.; ^*e*^Physiology Research Center, Ahvaz Jundishapur University of Medical Sciences, Ahvaz, Iran.

**Keywords:** TLR4, Morphine, Ibudilast, Inflammation, Innate immunity

## Abstract

**Background:**

Opioid drugs are used in the treatment of acute post-surgical pain and chronic pain, such as those associated with cancer. Opioid used is associated with complications such as analgesic tolerance, dependence and opioid abuse. The molecular mechanisms of unwanted opioid responses are varied but recent advances have highlighted elevations in pro-inflammatory cytokines and pro-inflammatory glial following chronic administration of morphine. In this study we investigated the neurodegenerative effects of morphine through its effects on Toll-Like Receptor 4 (TLR4) in the male rat hippocampus and evaluated the level of Interleukin-1 beta (IL-1β). Then we compared the difference between inhibitory effects on mu opioid receptors (by β-Funaltrexamine, β-FNA) and TLR4 (by Ibudilast). Subsequently, we assessed the amount of IL-1β and the number of granular cells in male rat hippocampus.

**Methods:**

Adult male rats (n=24) were treated with sucrose, morphine, Ibudilast (7.5 mg/kg) and β-FNA (20 mg/kg) for 30 days. Their brains were isolated and hemisected with one hippocampus for granular cell and the other used for IL-1 β immunoblotting.

**Results:**

Data showed that Ibudilast suppresses IL-1 β expression significantly more than β-FNA. The granular cell count displayed significant differences.

**Conclusions:**

Our results suggested that Ibudilast can be used for controlling and treatment of morphine-induced CNS inflammations or traumatic conditions.

## Introduction

Opium poppy extracts have been used as a pain reliever since 3500 BC. Opioid drugs are used to relieve chronic pains such as cancers. So far, an effective alternative drug to opioids has not been discovered.^1^ Many studies have shown that chronic administration of opioids leads to complications such as addiction, tolerance, dependence and abuse. Morphine is widely used and abused.^2-4^ Addicted patients have complications such as chronic headaches, vascular problems, increase in pro-inflammatory cytokines, hyperalgesia, allodynia, central nervous inflammations and glia cells activation.^2-8^

IL-1β is the most significant pro-inflammatory cytokine which has increased in patients with neural degeneration including multiple sclerosis (MS), Alzheimer Disease (AD), Huntington and also Parkinson.^9,10^ In simulated animals with Alzheimer Disease, cerebrovascular injuries and traumatic brain, cytokines such as TNF-α, IL-1β and IL-6 could play important roles in hippocampus neurodegeneration.^9-12^

The glia cells are responsible for maintaining the stability of the brain and spinal cord. The glia cells have classic opioid receptors (Mu, Kappa and Delta) and also toll-like receptors (TLRs), especially the TLR4. TLR4 plays an important role in innate immune system for recognition of external pathogen factors or internal ligands.^13,14^

Recent studies indicate that morphine is able to activate TLR4.^7^ TLR4 has been found in glia cells, neural stem cells and premature cells, especially in Dentate Gyrus of hippocampus in mammals and humans.^7^ TLR4 activation in acute or chronic neurodegenerative conditions such as cerebrovascular injuries, brain damage, chronic stresses, toxins, and infections lead to the release and secretion of pro-inflammatory cytokines.^12,15^ IL-1β is a key mediator in cell death.^16^ Long-term release of IL-1β through glia cells can influence life, growth, synapse transmissions and the process of memory and hippocampus state. IL-1β can lead to inhibition of the proliferation and differentiation of precursors and progenitor stem cells in the hippocampus. ^15,16^ It can finally lead to apoptosis and decrease in active and functional granular cells. ^12,16^

TLR4 is reported also on vascular endothelial cells and contributions of these cells accompanied by astrocytes can regulate the blood-brain barrier (BBB).^17^ The permeability of BBB in CNS health is also very important: the TLRs activation leads to cerebral side effects.^11,17,18^

Ibudilast is a nonspecific phosphodiesterase (PDE4) inhibitor that is used as an anti-inflammatory and anti-asthma treatment.^19^ Ibudilast has been investigated as a reducer of asthma symptoms, hyperalgesia, allodynia and neurovascular dysfunctions.^19^

In this study we investigated the neurodegenerative effects of morphine through its effects on TLR4 in the male rat hippocampus and evaluated the level of IL-1β. Then we compared the difference between the inhibitory effects on mu opioid receptors (by β-Funaltrexamine, β-FNA) and TLR4 (by Ibudilast). Subsequently, we assessed the amount of IL-1β and the number of granular cells in male rat hippocampus.

## Methods

**Animals**

24 Adult male albino NMRI rats (Razi Institute, Iran) 200-250 gr. were kept in individual plastic cages in pairs (40 × 25 × 25 cm) with wood chip bedding in a room with a 12-hour light cycle (12:12 light-dark) maintained at 23°C (±3°C). Animals had free access to food pellets and tap water^20^ for thirty days. Keeping the animals was in accordance with the standards of the committee of ethics on animal experiments at Ahvaz Jundishapour University of Medical Sciences.

**Chronic morphine administration**

Rats were made dependent by chronic administration of morphine sulfate (Temad, Iran) at doses of 0.1, 0.2 and 0.3 mg/ml each for 48 hrs, and 0.4 mg/ml up to 30 days. Sucrose (4 g/100ml) was added to drinking water to mask the bitter taste of morphine.^21^ In the control group A (n=6), rats were administrated sucrose (4g/100ml) in drinking water for the same duration of time. The withdrawal syndrome was precipitated by naloxone intraperitoneally (4mg/Kg, i.p.).^21^

**Experimental groups**

Group B (n=6): Rats were made dependent by chronic administration of morphine sulfate in drinking water as previously described.

Group C (n=6): Rats were made dependent by chronic administration of morphine sulfate and co-administrated with Ibudilast. Rats received Ibudilast (sigma) (7.5 mg/kg in 35% polyethylene glycol (PEG; sigma) in saline twice a day intraperitoneally (i.p).^22^

Group D (n=6): Rats were made dependent by chronic administration of morphine sulfate and co-administrated with β-Funaltrexamine (sigma). Rats received β-Funaltrexamine (20 mg/kg) twice a day intraperitoneally (i.p).^23^

On the 31st day, one hour after finishing experimental protocols (see above), all rats (n = 24) were anesthetized with diethyl ether and transcardially were perfused with 100 ml of saline followed by 100 ml fixative solution [paraformaldehyde (PFA) %4 in 0.2 M buffer phosphate at pH = 7.4]. After perfusion, all rats were decapitated and their brains were removed. The whole hippocampi from their left hemispheres were dissected immediately at 4°C and snap-frozen in liquid nitrogen and then stored in -80°C until use. The right hemispheres were kept in 4% PFA for at least one week and were then processed for histological studies as follows. The coronal serial sections with 10-μm thickness were cut from right hippocampus.^24^ We chose one from each 10 sections and all were stained with Toluidine Blue 1%. Slides were examined with a light microscope and digital photographs were taken from hippocampal dentate gyrus areas in right hemispheres.^25^ Cell count was done in a 25×25 µm^2^ field in dentate gyrus areas. Then, the number of cells in a unit volume was calculated.^26^

**Protein extraction, gel electrophoresis and staining**

Snap-frozen samples were homogenized individually in lysis buffer (150 mM Tris (pH 6.8), 12% (w/v) SDS, 36% (v/v) glycerol, and 6% (v/v) 2-mercaptoethanol). The samples were centrifuged (15,000 × g, 30 min, 4°C), and the supernatants were stored at -20°C.^26^ For the analysis of the target proteins 100 ± 10 mg of hippocampus was homogenized (60s) with an ultra-sonic homogenizer in 5 volumes of 10 mM Tris HCl bufer, pH 7.4, containing 150 mM NaCl, 0.03% Nonidet P-40 (NP-40), and the following protease and phosphatase inhibitors. The samples were then sonicated (10 s) and centrifuged at 4°C and 15,000g for 15 min. The supernatant was recovered and the protein content determined by the Bradford protein assay protocol with bovine serum albumin as the standard. An aliquot (400 µl) of the supernatant was mixed with 50 µl of 160 mM Tris HCl, 8% SDS, pH 6.8, and 50 µl of electrophoresis loading buffer (500 mM Tris HCl, 8% SDS, 30% glicerol, 20% 2- mercaptoethanol, 0.02% bromophenol blue, pH 6.8) and boiled. The samples were then submitted to SDS-PAGE in a 16% gel (1.5 mm thickness). Protein bonds were revealed by coomassie brilliant blue R-250 staining and destaining was done using methanol and acetic acid for 3 hours.^27^ The molecular weight of each band was identified by the standard proteins using the following molecular weights: Bovine Serum Albumin (66kDa); Ovalbomin (44kDa); Carbonic Anhydrase (29kDa); Myoglobin (17kDa) and lysosyme (14kDa).

**Pro-inflammatory cytokine qualification**

After protein extraction as described in the previous section,proteins were transferred to 0.45 micron (for IL-1β immunoblotting) PVDF membranes and blocked at room temperature for 1 hour with phosphate buffered saline solution (PBS in mM: NaCl 137, KCl 2.7, Na2HPO4 12, KH2PO4 1.38, pH 7.4) containing 5% nonfat dry milk, 0.5% bovine serum albumin and 0.2% Tween 20 (blocking solution).^28^ Then, the membrane was incubated overnight at 4°C in blocking solution containing the primary antibody: anti-IL-1β dilution of 1: 500 (Santa Cruz Biotechnology, CA, U.S.A.). The secondary antibody, horseradish peroxidase-linked goat anti-rat immunoglobulin G (IgG) (Santa Cruz Biotechnology, CA, U.S.A.) was incubated at 1: 6000 dilutions in blocking solution at room temperature for 2 hours. Immunoreactivity of IL-1β protein was detected with the brown 3, 3’-Diaminobenzidine (DAB). The molecular weight of each band was identified by the standard proteins with the following molecular weights: Bovine Serum Albumin (66kDa); Ovalbomin (44kDa); Carbonic Anhydrase (29kDa); Myoglobin (17kDa) and lysosyme (14kDa).

**Histological studies**

Right hemispheres were processed, paraffin embedding was done and they were cut in to slices of 10 micrometer thickness. Slides were stained by Toluidine Blue 1% in order to count the number of granular cells in dentate gyrus of right hippocampal slices. After light microscopic photography (objective 40X) from stained slides, we compared the granular cell numbers between the groups. 

**Stereological and Biometrical Procedures**

Counts of granular cell bodies (somata) appearing in the dentate gyrus layer of the 10 micron-thick Nissl- stained sections were done by using a 40X objective and following a simplified version of the stereological Optical Fractionators’ method. In short, cell somata were counted in the sampling area. Cells appearing in the upper focal plane were omitted to prevent overcountings. Counts were systematically done at sampling points during sequential movements of 25x25 microns in 100x100 areas in the X-Y plane along the 10 micron-thick section. The estimation of the total number of cells in the layer was calculated according to formula by West et al. (1991) :^28^

$$ N= \Sigma Q^-\cdot \frac{t}{h} \cdot \frac{1}{asf} \cdot \frac{1}{ssf}  $$

Total neuronal quantity (**N**) for right hippocampal dentate gyrus, ** [ΣQ]-** is the total quantity of neurons counted in the dissectors on the sampled sections,** ssf ** is Section sampling fraction was 1/10, since each tenth section was used for analysis, **asf** is area sampling fraction (25x25 / 100x100), **t** is the mean thickness of the sections (μm) and **h** is the optical dissector height (μm), (t/h =19). Means were determined for each experimental group. The mean number of sampling points in every rat hippocampus varied from 20 in group A, 13 in group B, 18 in group C and 15 in group D. Biometrical measures of cell somata were done in Nissl stained preparations from all groups’ specimens, at least forty photographs from each group, obtained with an Olympus Dp-70 camera. The different parameters evaluated were measured with the appropriate software.^26,28,29^

**Statistical analysis**

The data were statistically analyzed using SPSS 11.5 software. Multiple comparisons were performed by one way analysis of variance test (ANOVA). To determine the difference between groups the analysis was followed by Post Hoc TUKEY. Significance was established when the probability of values were less than or equal to 0.05.

## Results

**Protein extraction and IL-1β**

Our data showed polymorphism is the basis of difference in protein intensity among treatments. The most diversity (added or removed bands) was observed in areas with 14-29 kDa and 42-46 kDa molecular weights.

**Diagram 1:**
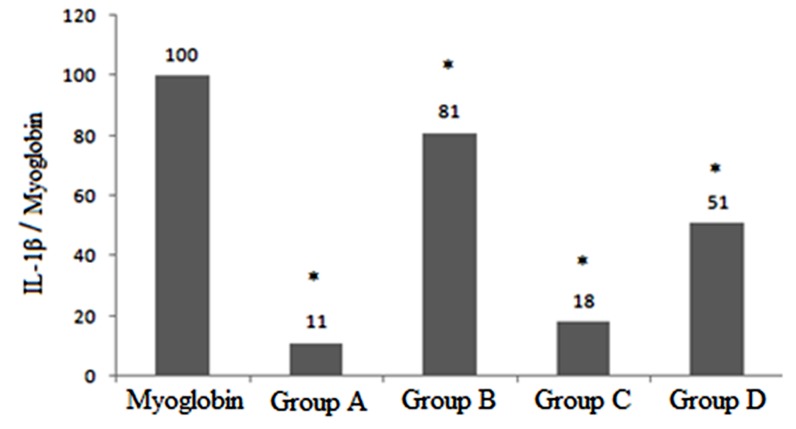
IL1-β and myoglobin (M) as standard marker levels were quantified by densitometry. Data are presented as mean ± S.E. and expressed as percentage with respect to normalized myoglobin level as a standard marker (defined as 100%). (٭= significant difference P ≤ 0.0). Group A: control group; Group B: chronic morphine; Group C: chronic morphine + Ibudilast; Group D: chronic morphine + β-Funaltrexamine.

Diagram 1 is presented to determine the inhibitory effects of drugs on the release of IL-1β in hippocampus of the treated animals. 

We studied the influence of mu opioid receptors (MORs) and Toll- Like Receptor 4 (TLR4) on releasing IL-1β in the hippocampus and then compared their effects.

**Figure 1:**
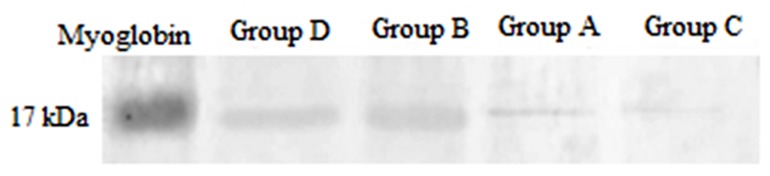
Rat hippocampus ILl-1β, a representative western blot is shown. Group A: control group; Group B: chronic morphine; Group C: chronic morphine + Ibudilast; Group D: chronic morphine + β-Funaltrexamine.

The western blot showed that the IL-1β expressions in group B were elevated and more expressed compared with group A (p<0.05) and this was the highest expression in all groups. The IL-1β expression in group C was suppressed compared with group B (p<0.05). On the other side, our data indicated that the IL-1β in group C did not show any significant difference comparing with group A. Our data showed that IL-1β was suppressed in group C compared with group B (p<0.05). (Diagram 1)(Figure 1).

**Granular Cell Count**

**Diagram 2:**
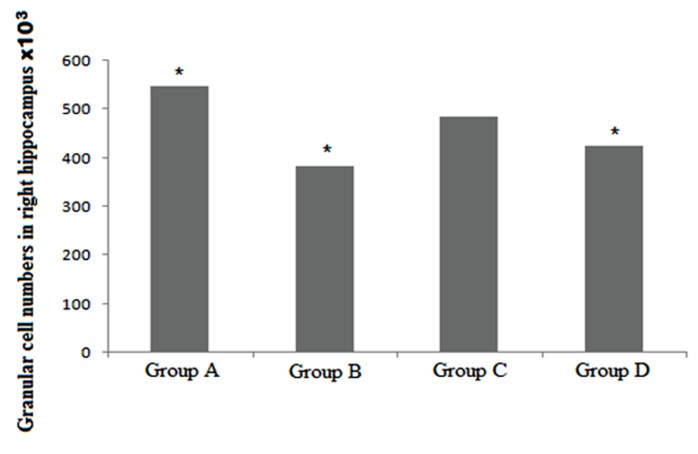
Granular cell numbers in right hippocampus. Greatest significant reduction between all groups is in group B. There are no significant difference between control group A and group C (Iibudilast treated). (* = P < 0.05). Group A: control group; Group B: chronic morphine; Group C: chronic morphine + Ibudilast; Group D: chronic morphine + β-Funaltrexamine.

**Figure 2:**
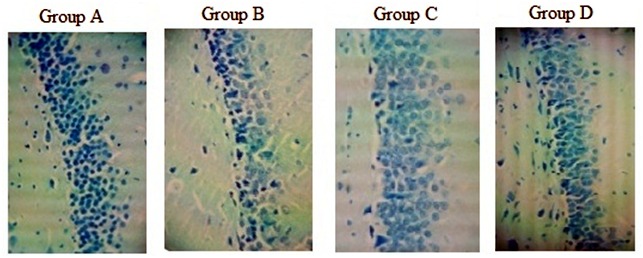
Dentate gyrus of rat’s hippocampus shows the difference in granular cells crowd between groups due to drugs treatment. Group A: control group; Group B: chronic morphine; Group C: chronic morphine + Ibudilast; Group D: chronic morphine + β-Funaltrexamine.

The cell counts showed that we had significant reduction in group B [F (3,156) = 20.867, (P <0.05)], the smallest count. Data showed that chronic morphine (group B) induced about 30% reduction in the number of granular cells compared with group A (P <0.05). There was a significant difference between Ibudilast treated (group C) and chronic morphine (group B) (P <0.05) (Diagram 2). There was no significant difference between group C and group A. Our results showed that Ibudilast improved the morphine complication compared with β-FNA. Representative photomicrographs of hippocampal sections show Nissl-staining for the different groups(Figure 2).

## Discussion

Morphine can stimulate the immune cells in CNS like microglia and astrocytes via TLR4. These cells can release important mediators which are able to change the normal conditions in neurogenic microenvironment in the hippocampus.^4,30,31^

Ibudilast has a good permeability from the blood-brain-barrier.^32^ It is documented that Ibudilast can suppress the pro-inflammatory mediators released by microglia in vitro and in vivo.^32^ It is a non-specific phosphodiesterase inhibitor. It can suppress the production of pro-inflammatory cytokines and chemokines, nitric oxide.^32^ It can also enhance the quality and duration of morphine analgesia.^32,33^

Morphine-induced glial pro-inflammatory cytokines release responses have been documented to improve tolerance to opioid analgesia.^34,35^ Astrocytes and microglia respond to chronic administration of morphine in a pro-inflammatory pattern, with up-regulated activation and the release of pro-inflammatory cytokines like IL-1β.^4,5^ Suppressing and blocking pro-inflammatory cytokines releasing from glia, or genetically diminishing interleukin-1 pathways and signaling significantly reduces and delays morphine tolerance, dependency and addiction side effects.^31^ Chronic administration of morphine leads to impairment of learning and memory process.^36^

Chronic morphine intake induces stimulation and activation of glia cells to release and secretion of pro-inflammatory cytokines. In this regard, no difference was observed between our results and other studies performed on the effects of morphine. Many researchers attribute these complications to the relationship between morphine and a classic receptor named Mu opioid receptor (MOR).^36^ In this study, we found that Ibudilast suppresses IL-1 expression significantly more than β-FNA. Data showed that inhibition of TLR4 is significantly more powerful than MORs to prevent pro-inflammatory release in adult rat hippocampus.

Release of IL-1β from stimulated glia cells by morphine can inhibit proliferation and differentiation of neural stem cells in hippocampus. Subsequently it can suppress the neurogenesis in sub-granular zone (SGZ).^30^

In this study comparison of results between groups under simultaneous treatment of chronic self-administration of morphine and irreversible MOR Antagonist (beta-Funaltrexamine, β-FNA) or Ibudilast showed the maximum rate of activity in favor of pro-inflammatory cytokines is caused by the activation of glia cells by mediating TLRs. IL-1β values in these groups indicate significant and valuable differences between the inhibition of the TLRs or MORs. The inhibitory effects of Ibudilast on provoked TLRs through non-specific diphosphoesterase inhibition lead to reduction in release of IL-1β and reduced complications in dentate gyrus of the hippocampus. Morphine intake leads to release of IL-1β in group B at a higher level than other groups. Simultaneous administration of morphine with irreversible MORs antagonist (β-FNA) or using morphine and Ibudilast as a TLR4 inhibitor drug lead to the significant reduction and variation in IL-1β. 

The present study, along with other studies performed on hippocampus histology using Nissl- staining (Toluidine Blue1%), showed that granular cell counts in dentate gyrus of animals in group C (Ibudilast + morphine) are near to normal for animals in control group (A) and there is a significant difference (P<0.05) between group D (β-FNA + morphine) and control group (A). It means that TLR4s are more powerful than MORs in causing degenerative conditions. Activated TLR4 also deregulate neurogenic environments which lead to cell death and apoptosis.

Having considered all the above mentioned evidence, it can be concluded that chronic administration of morphine (in cancer patients or addicted people) can cause side effects such as cerebral inflammation, cerebrovascular injuries, severe pains, (Hyperalgesia & Allodynia) and even chronic degenerative diseases.

We suggest that Ibudilast is used during treatment of morphine-induced chronic cerebral inflammation.
